# Emerging applications of NLP and large language models in gastroenterology and hepatology: a systematic review

**DOI:** 10.3389/fmed.2024.1512824

**Published:** 2025-01-22

**Authors:** Mahmud Omar, Salih Nassar, Kassem SharIf, Benjamin S. Glicksberg, Girish N. Nadkarni, Eyal Klang

**Affiliations:** ^1^Maccabi Health Services, Tel Aviv, Israel; ^2^Division of Data-Driven and Digital Medicine (D3M), Icahn School of Medicine at Mount Sinai, New York, NY, United States; ^3^Edith Wolfson Medical Center, Holon, Israel; ^4^Department of Gastroenterology, Sheba Medical Center, Tel HaShomer, Israel

**Keywords:** natural language processing, large language models, gastroenterology, hepatology, electronic health records

## Abstract

**Background and aim:**

In the last years, natural language processing (NLP) has transformed significantly with the introduction of large language models (LLM). This review updates on NLP and LLM applications and challenges in gastroenterology and hepatology.

**Methods:**

Registered with PROSPERO (CRD42024542275) and adhering to PRISMA guidelines, we searched six databases for relevant studies published from 2003 to 2024, ultimately including 57 studies.

**Results:**

Our review of 57 studies notes an increase in relevant publications in 2023–2024 compared to previous years, reflecting growing interest in newer models such as GPT-3 and GPT-4. The results demonstrate that NLP models have enhanced data extraction from electronic health records and other unstructured medical data sources. Key findings include high precision in identifying disease characteristics from unstructured reports and ongoing improvement in clinical decision-making. Risk of bias assessments using ROBINS-I, QUADAS-2, and PROBAST tools confirmed the methodological robustness of the included studies.

**Conclusion:**

NLP and LLMs can enhance diagnosis and treatment in gastroenterology and hepatology. They enable extraction of data from unstructured medical records, such as endoscopy reports and patient notes, and for enhancing clinical decision-making. Despite these advancements, integrating these tools into routine practice is still challenging. Future work should prospectively demonstrate real-world value.

## Introduction

Recent advances in Natural Language Processing (NLP) show potential for being integrated in the field of gastroenterology and hepatology (1, 2). Since the last review in 2014 by Hou et al.—which highlighted NLP’s growing utility in gastroenterology, particularly for extracting structured data from colonoscopy and pathology reports to track quality metrics and improve disease detection (2)—the field has evolved considerably. The earlier work by Hou et al. demonstrated promising performance in relatively focused domains, such as colonoscopy quality measure extraction and improving case-finding for inflammatory bowel disease, yet it largely described proof-of-concept implementations and noted challenges with integration into routine clinical workflows and data heterogeneity across settings.

In contrast, significant strides in technology, including the advent of Large Language Models (LLMs) such as Generative Pre-trained Transformer (GPT) and Bidirectional Encoder Representations from Transformers (BERT) (3), have expanded the scope of NLP applications. While Hou et al.’s era of NLP research centered on rule-based or traditional machine learning methods optimized for specific tasks, newer LLMs can handle a broader range of complex and context-rich functions, from automating routine documentation tasks to supporting sophisticated diagnostic reasoning and therapeutic decision-making (4). These contemporary models may better address scalability and integration challenges, moving beyond static data extraction toward dynamic interactions with unstructured clinical narratives.

NLP and LLMs extract and interpret data from patient records, notes, and reports (5–7). In gastroenterology and hepatology, they streamline the review of endoscopy, radiology, and pathology reports. This technology can help create research cohorts for clinical trials, flag complications, and support decision-making systems. Examples include managing complex conditions like IBD and hepatocellular carcinoma (5, 7, 8).

This review discusses the current applications and challenges of NLP and LLMs in gastroenterology and hepatology.

## Methods

### Registration and protocol

This systematic literature review was registered with the International Prospective Register of Systematic Reviews, PROSPERO, under the registration code CRD42024542275 (9). Our methodology adhered to the Preferred Reporting Items for Systematic Reviews and Meta-Analyses (PRISMA) guidelines (10).

### Search strategy

We conducted a systematic search of six key databases (PubMed, Embase, Web of Science, and Scopus, Cochrane library and IEEE Xplore) for studies published until April 2024. Our focus was on the outcomes of integrating NLP and LLM models in gastroenterology and hepatology. We designed Boolean search strings tailored to each database. To maximize coverage, we supplemented our search with a manual reference screening of included studies and targeted searches on Google Scholar. Details of the specific Boolean strings used are provided in the Supplementary materials.

### Study screening and selection

Our review encompasses original research articles, and full conference papers (11). The exclusion criteria were confined to preprints, review papers, case reports, commentaries, protocol studies, editorials, and non-English publications. For the initial screening, we used the Rayyan web application (12). The initial screening and study selection, which were conducted according to predefined criteria, were independently performed by two reviewers (MO and EK). Discrepancies were resolved through discussion. Fleiss’ kappa was calculated for the agreement between the two independent reviewers.

### Data extraction

Data extraction was conducted by researchers MO and EK using a standardized form to ensure consistent and accurate data capture. This included details such as author, publication year, sample size, data type, task type, specific field, model used, results, numeric metrics, conclusions, and limitations. Any discrepancies in data extraction were resolved through discussion and a third reviewer was consulted when necessary.

### Risk of bias assessment

To ensure a thorough evaluation of the included studies, we used three tools, each tailored to a specific study design within our review. The Risk Of Bias In Non-randomized Studies of Interventions (ROBINS-I) tool has been employed in interventional studies assessing NLP in applications such as management, prescription guidance, and clinical inquiry responses (13). For diagnostic studies where NLP models were compared with physicians or a reference standard for diagnosing and detection, the Quality Assessment of Diagnostic Accuracy Studies-2 (QUADAS-2) tool was used (14). Finally, the Prediction model Risk Of Bias ASsessment Tool (PROBAST) tool was utilized for the remaining studies, which involved NLP models prediction, without direct comparison to reference standards (15). This multitool approach allowed us to appropriately address the diverse methodologies and applications presented in the reviewed studies.

## Results

### Search results and study selection

**Figure 1:**
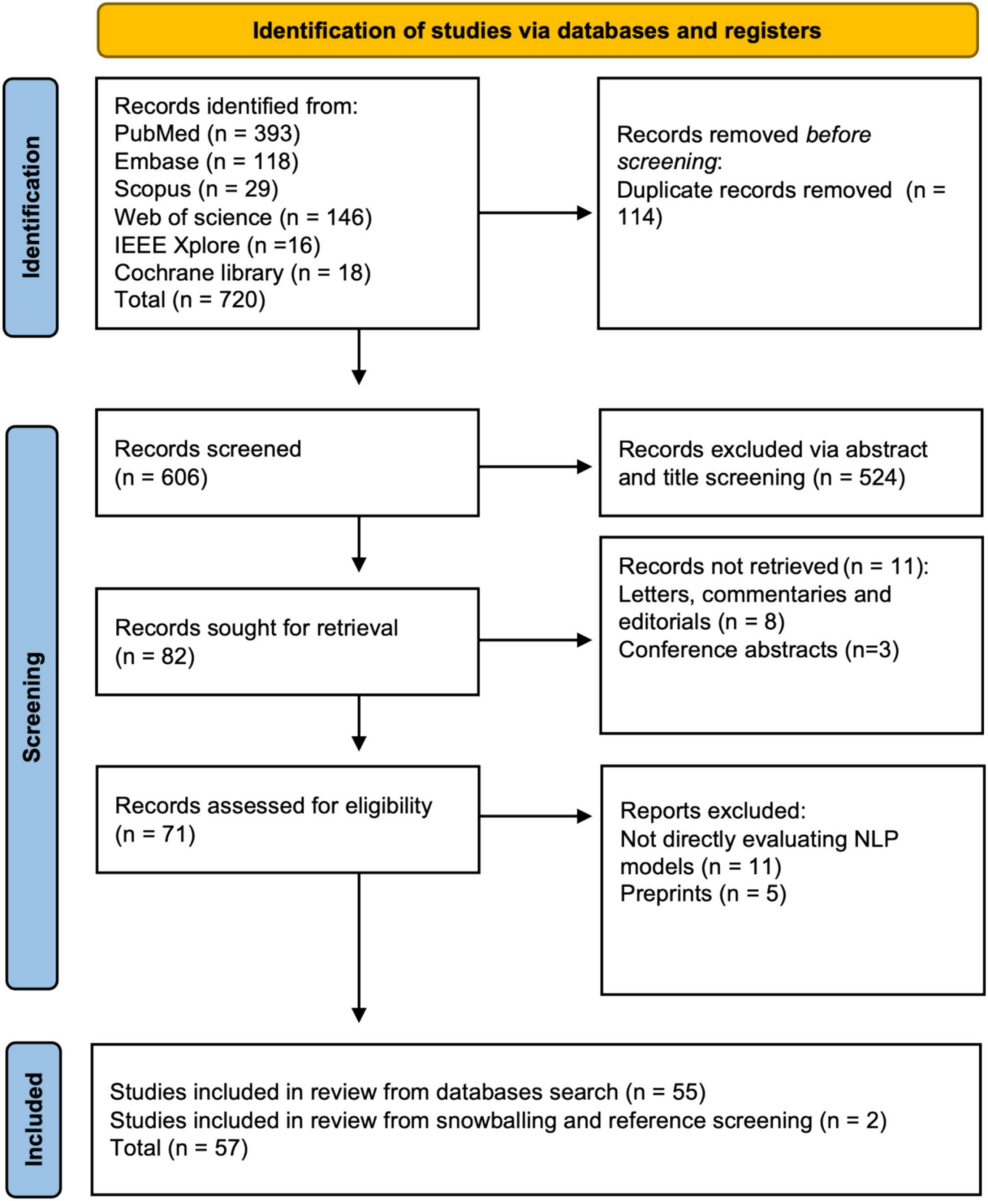
PRISMA flowchart.

A total of 720 articles were identified through initial screening. After the removal of 114 duplicates, 606 articles remained for further evaluation. Title and abstract screening led to the exclusion of 524 articles, leaving 82 articles for full-text review. Of these, the reasons for exclusion and the number of articles excluded for each reason remain the same as described earlier. Ultimately, 55 studies met all inclusion criteria. By employing reference checking and snowballing techniques, two additional studies were identified, resulting in a final tally of 57 studies (16–72). A PRISMA flowchart visually represents the screening process in Figure 1. Fleiss’ kappa for the agreement between screeners was calculated as 0.957, which is considered very high (73).

### An overview of the included studies

Our systematic review incorporates a total of 57 studies (16–72). Among these, a substantial majority, 49 studies, are centered on gastroenterology, while hepatology is the focus of 8 studies. These studies span from 2018 to 2024, with a notable increase in publications in the last 2 years, particularly between 2023 and 2024, which collectively account for 28 of the total included studies. This uptick highlights a growing interest in advanced NLP models like GPT-3 and GPT-4.

The models employed in these studies vary widely, with traditional NLP methods and more recent LLMs like GPT-3 and GPT-4. For instance, Kong et al. (2024) utilized GPT-4 among other versions for medical counseling (38), while Schneider et al. (2023) employed rule-based NLP algorithms for detecting undiagnosed hepatic steatosis (54).

Sample sizes in these studies range from very small datasets to large-scale analyses involving millions of data points, such as in the study by Schneider et al., which analyzed data from over 2.7 million imaging reports (54). The type of data analyzed also varies significantly, encompassing electronic health records (EHRs), pathology reports, and data generated from AI models responding to preset medical queries.

Tasks performed by these models are equally diverse, from diagnostic assistance and disease monitoring to providing patient education and supporting clinical decision-making. Specific examples include the work by Truhn et al. (2024), which focused on extracting structured data from colorectal cancer reports (49), and Lahat et al. (2023), who evaluated the utility of GPT models in answering patient questions related to gastroenterology (47).

### Risk of bias

**Figure 2:**
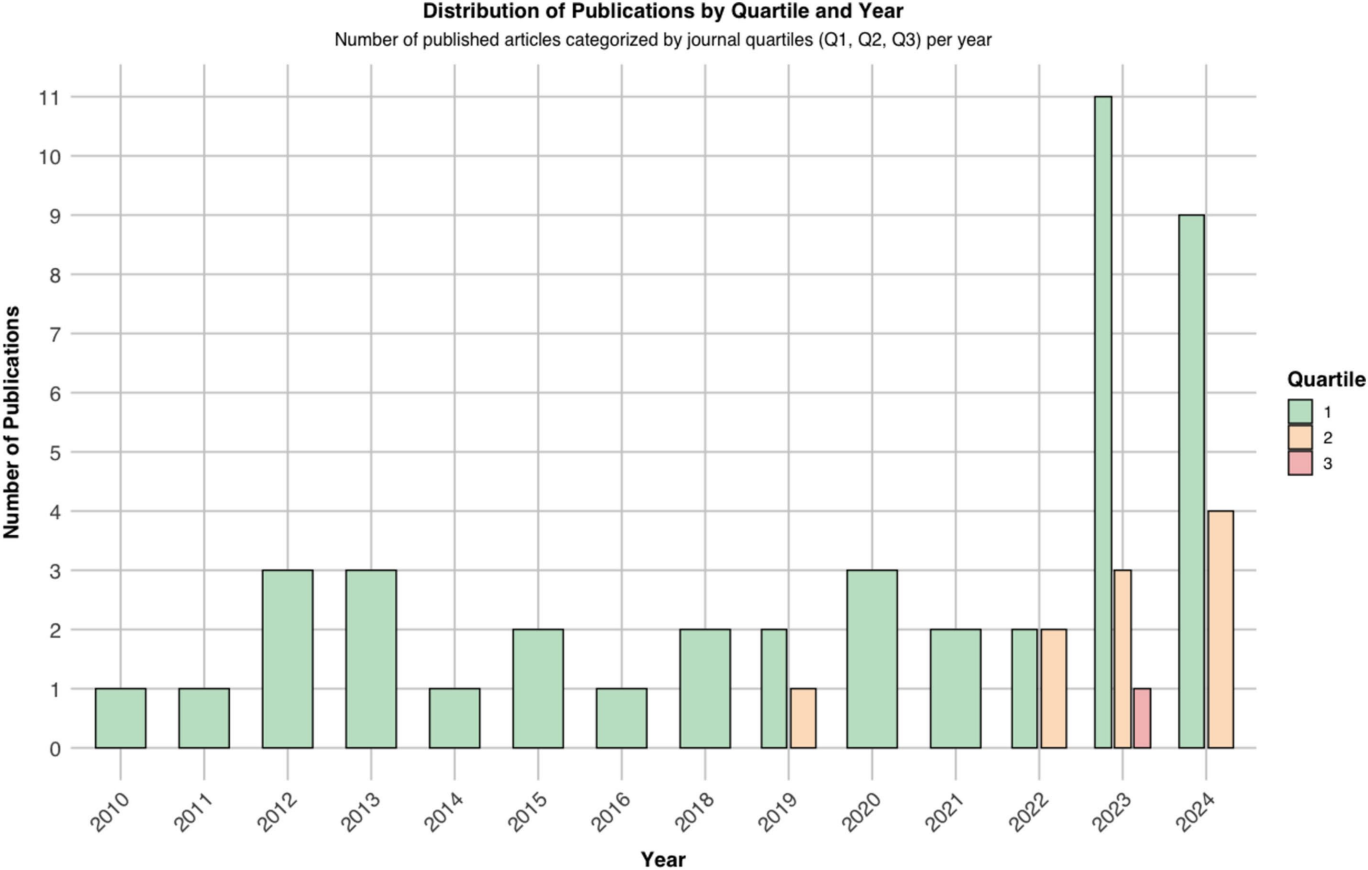
Trends of the included studies.

We used ROBINS-I, QUADAS-2, and PROBAST to map potential biases. Notably, most of the included studies were published in Q1 journals, affirming their scholarly impact and supported by strong SCImago Journal Rank (SJR) scores (Figure 2).

#### PROBAST results (Supplementary Table S1)

This assessment mostly highlighted low-risk ratings in outcome and analysis domains. However, several studies encountered issues with high participant-related applicability biases, influencing the generalizability of their findings.

#### QUADAS-2 results (Supplementary Table S2)

A synthesis of QUADAS-2 results revealed that most studies (20 out of 32) exhibited low risk of bias across all four assessed domains. This underscores their methodological robustness and reliability. However, three studies were identified as having a high risk of bias in one of the four categories. Patient selection applicability concerns were notable, primarily due to the reliance on single-center data with specific documentation styles, which may limit the broader applicability of these findings.

#### ROBINS-I results (Supplementary Table S3)

Analysis of ROBINS-I revealed that 14 studies displayed a moderate risk of bias overall, while one study exhibited a high risk. This was largely due to biases in the selection of participants into the study and confounding factors, particularly because many studies utilized specific questions, queries, or fictional vignettes and case scenarios. Despite these concerns, the other assessment categories predominantly showed low risk. Nonetheless, six studies demonstrated low risk across all evaluated domains.

### NLP applications

**Figure 3:**
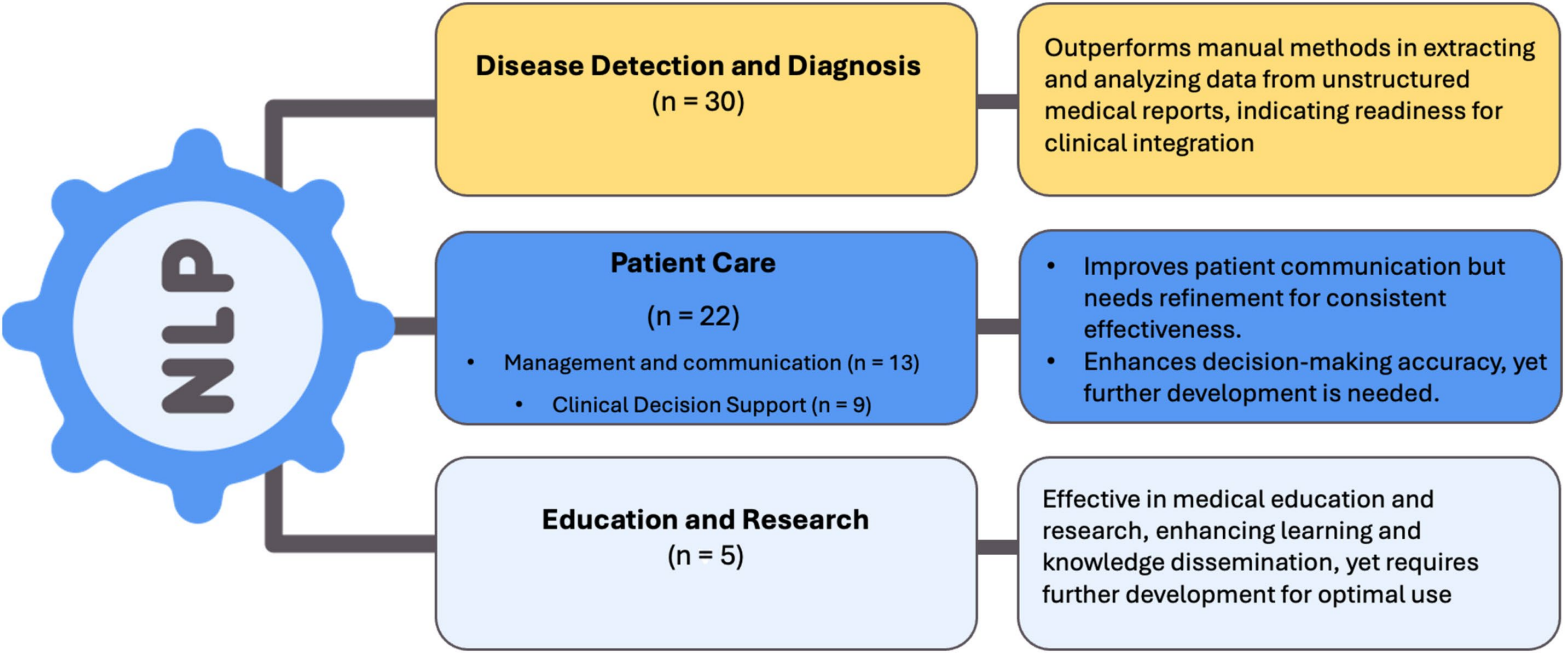
Summary of NLP applications and outcomes.

We categorized the applications of the NLP and LLM models under three main categories for a synthesized analysis of the results: Disease Detection and Diagnosis (*n* = 30), Patient Care (*n* = 22), and Education and Research applications (*n* = 5). Disease Detection and Diagnosis was further divided into Colonoscopy Reports and Other Diagnostic Applications, recognizing that digitized pathology reports—though ultimately part of the broader EHR—were considered separately to better capture unique NLP tasks. Patient Care was subdivided into Management and Communication and Clinical Decision Support, focusing on patient-centered and healthcare professional–oriented applications, respectively (Tables 1, 2 and Figure 3).

**Table 1:** Summary of the included studies.

Author	Year	Data Type + Sample size	Model	Model Task	Main Result
Gastroenterology					
Kong et al. (38)	2024	15 questions related to *H. pylori*	ChatGPT 4.0, ChatGPT 3.5, ERNIE Bot 4.0	Counseling on *H. pylori* infection	ChatGPT 4.0 achieved 90% accuracy in responses and 100% comprehensibility but had a lower completeness rate at 45.6%. ChatGPT 3.5 had an accuracy of 88% and a completeness rate of 40.9%, while ERNIE Bot 4.0 showed lower scores across all metrics.
Lahat et al. (46)	2023	110 real-life patient questions	GPT	Answering patient questions	ChatGPT’s accuracy varied across question types, with a mean accuracy score ranging from 3.4 to 3.9 out of 5. It performed better in treatment-related questions (average score: 3.9) compared to diagnostic questions (average score: 3.4).
Truhn et al. (49)	2024	100 colorectal cancer reports	GPT-4	Extracting structured information	GPT-4 achieved 99% accuracy in extracting T-stage, 95% for N-stage, and 94% for M-stage from unstructured histopathology reports.
Zhou et al. (48)	2023	23 medical knowledge questions	GPT-3.5 and GPT-4	Gastric cancer consultation and report analysis	GPT-4 achieved 91.3% appropriateness and 95.7% consistency in a gastric cancer knowledge test. GPT-3.5 had 73.9% appropriateness and 82.6% consistency.
Choo et al. (39)	2024	30 patients with Stage IV or recurrent colorectal cancer	GPT	Formulating management plans	ChatGPT achieved an 86.7% concordance with the Multidisciplinary Tumor Board decisions, including a 73.3% level 1 concordance for first-line treatments.
Huo et al. (44)	2024	Responses for 9 patient cases	ChatGPT, Bing Chat, Google Bard, Claude 2	Providing screening recommendations	ChatGPT aligned with guidelines in 77.8% of clinician cases and 55.6% of patient cases. Bing Chat, Google Bard, and Claude 2 had alignment rates ranging from 25 to 66.7%.
Imler et al. (58)	2013	500 colonoscopy and pathology reports	cTAKES NLP engine	Categorizing pathology findings	The NLP engine achieved 98% accuracy in identifying pathology levels, 97% accuracy for location, and 84% accuracy for the number of adenomas.
Lim et al. (33)	2024	62 example case scenarios, tested three times	GPT-4, contextualized and non-contextualized	Providing advice on colonoscopy intervals	The contextualized GPT-4 model identified high-risk features with 79% accuracy and recommended correct colonoscopy intervals 79% of the time, compared to 51% with the standard model.
Imler et al. (36)	2014	10,798 colonoscopy reports, 6,379 linked to pathology	Clinical text analysis and knowledge extraction system (cTAKES)	Determining colonoscopy surveillance intervals	Achieved an agreement level of 81.7% with manual review, with a Pearson R of 0.813.
Bae et al. (60)	2022	2,425 colonoscopy and pathology reports	Regular expressions and smartTA	Assessing quality indicators	The NLP pipeline achieved 99–100% accuracy for identifying polyp subtypes, anatomical locations, and neoplastic polyps.
Denny et al. (63)	2012	200 patients	KnowledgeMap Concept Identifier	Identifying colorectal cancer tests in EMRs	Achieved 93% recall and 94% precision in identifying CRC tests, outperforming manual reviews (74% recall).
Lahat et al. (47)	2023	20 research questions	GPT	Generating gastroenterology research questions	The model generated relevant questions with a mean clarity score of 4.6 but had low originality (1.5 out of 5).
Laique et al. (26)	2021	35,914 colonoscopy reports	Optical Character Recognition (OCR) and NLP	Extracting quality metrics	Achieved over 95% accuracy for various clinical variables, with some metrics exceeding 99%.
Blumenthal et al. (65)	2015	1,531 patients	NLP tool called QPID	Predicting non-adherence to colonoscopy	Achieved an AUC of 70.2%, with 92% specificity and a PPV of 26%.
Harkema et al. (42)	2011	679 colonoscopy and pathology reports	Rule-based NLP engine	Quality measurement in colonoscopy	Achieved an F-measure of 0.74 and accuracy of 0.89 for various quality metrics.
Raju et al. (59)	2015	12,748 colonoscopy patients	Custom NLP software	Reporting colonoscopy quality metrics	Achieved 91.3% accuracy in identifying screening colonoscopies and 99.4% accuracy in adenoma identification.
Nayor et al. (57)	2018	8,032 screening colonoscopies	NLP pipeline	Calculating adenoma and serrated polyp detection rates	Achieved 100% precision and recall for both adenomas and serrated polyps.
Atarere et al. (25)	2024	20 questions using AI models	ChatGPT, BingChat, and YouChat™	CRC screening advice	Achieved 89.2% inter-rater reliability with variable alignment to clinical guidelines.
Seong et al. (40)	2023	280,668 colonoscopy reports	LSTM, BioBERT, Bi-LSTM-CRF	Extracting information from reports	Bi-LSTM-CRF achieved F1 scores from 0.9564 to 0.9862 across various findings.
Lee et al. (21)	2019	800 colonoscopy reports	Commercial NLP tool	Identifying quality and large polyps	Achieved 100% sensitivity and a PPV of 90.6% for identifying large polyps.
Denny et al. (50)	2010	200 patients	KnowledgeMap concept identifier	Detecting colonoscopy timing and status	Achieved a recall of 0.91 and precision of 0.95 for timing descriptors.
Parthasarathy et al. (18)	2020	323,494 colonoscopy patients	NLP	Diagnosing serrated polyposis syndrome	Achieved 93% accuracy in identifying correct SPS diagnoses.
Rammohan et al. (68)	2024	NR	GPT-4 and Bard	Answering standard gastroenterology questions	ChatGPT 4.0 achieved a mean reliability score of 6.23, while Bard had a mean of 2.04.
Pereyra et al. (37)	2024	238 physicians	GPT-3.5	Assessing CRC screening recommendations	ChatGPT had a mean score of 4.5/10, compared to 7.71/10 for physicians with the app.
Song et al. (61)	2022	1,000 validation, 248,966 application EGD reports	Custom NLP pipeline	Extracting information from EGD reports	Achieved sensitivity, PPV, accuracy, and F1 scores above 0.966 for various conditions.
Peng et al. (45)	2024	131 colorectal cancer questions	GPT-3.5	Answering CRC-related questions	Achieved a mean accuracy score of 0.91, but lower comprehensiveness (0.85).
Tinmouth et al. (70)	2023	1,450 pathology reports	NLP	Identifying adenomas for ADR	Achieved sensitivity of 99.60% and specificity of 99.01%.
Mehrotra et al. (27)	2012	24,157 colonoscopy reports	NLP (C-QUAL)	Assessing colonoscopy quality measures	Achieved kappa >0.7 for nine out of 20 measures.
Becker et al. (64)	2019	2,513 German clinical notes from 500 patients	German-specific NLP pipeline	Guideline-based treatment evaluation	Achieved 96.64% precision and 94.89% recall for tumor stage detection.
Hou et al. (31)	2013	575 colonoscopy pathology reports	Automated Retrieval Console (ARC)	Identifying surveillance colonoscopy	Achieved 77% recall and 80% precision for surveillance reports.
Gorelik et al. (51)	2023	20 clinical scenarios	GPT-4	Post-colonoscopy patient management	Achieved 90% compliance with guidelines and an 85% accuracy in recommendations.
Samaan et al. (34)	2023	91 questions on liver cirrhosis	GPT	Answering cirrhosis-related questions in Arabic	Achieved 72.5% accuracy in Arabic, with comprehensive responses only in 24.2% of cases.
Cankurtaran et al. (67)	2023	20 questions on Crohn’s disease and ulcerative colitis	GPT	Responding to IBD queries	Scored higher for professional queries (mean reliability: 6/7) than for patient queries (mean: 4/7).
Nguyen Wenker et al. (69)	2023	1,000 patients for NLP validation	CLAMP NLP software	Identifying dysplasia in Barrett’s Esophagus	Achieved 98.7% accuracy, 100% precision, and 92.3% recall.
Imler et al. (66)	2018	23,674 ERCP procedures	NLP	Quality measurement for ERCP	Achieved accuracy of 90–100% and precision of 84–100%.
Li et al. (62)	2021	5,570 patients	NLP	Identifying Lynch Syndrome for MMR screening	Achieved 100% sensitivity, specificity, PPV, and NPV.
Li et al. (16)	2022	22,206 patients across various tests	ENDOANGEL-AS NLP and deep learning	Identifying high-risk patients for surveillance	Achieved 100% accuracy in internal testing and 99.91% in external testing.
Wagholikar et al. (35)	2012	53 patients	NLP	Providing colonoscopy surveillance guidance	Made optimal recommendations in 90.6% of cases.
Sciberras et al. (20)	2024	38 questions from IBD patients	GPT-3.5	Generating responses to IBD patient queries	Achieved 84.2% accuracy with a median score of 4.0 for completeness.
Stidham et al. (52)	2023	1,240 patients with IBD	NLP	Detecting and inferring EIM activity status	Achieved 94.1% accuracy, with sensitivity of 0.92 and specificity of 0.95.
Ganguly et al. (22)	2023	2,276 colonoscopy procedures	NLP	Adenoma detection and report card generation	Achieved 100% sensitivity, specificity, and accuracy.
Ma et al. (43)	2024	165 esophageal ESD cases	GPT-3.5	Post-procedural quality control for esophageal ESD	Achieved accuracy of 92.5–100% across different factors.
Gravina et al. (32)	2024	Questions from 2023 Italian medical exam	GPT 3.5 and Perplexity AI	Answering medical residency exam questions	GPT 3.5 achieved 94.11% correct responses in the latest exam.
Fevrier et al. (84)	2020	401,566 colonoscopy linked with pathology reports	SAS® PERL NLP tool	Extracting data from colonoscopy reports	Achieved Cohen’s κ between 93 and 99% and PPV of 97–100% for common categories.
Benson et al. (55)	2023	24,584 pathology reports	NLP pipeline	Extracting features of colorectal polyps	Achieved 98.9% precision and 98.0% recall, with an F1-score of 98.4%.
Zand et al. (23)	2020	16,453 lines of dialog from 424 patients	NLP model	Developing a chatbot for IBD patient support	Achieved 95% agreement with physician evaluations in categorizing dialogs.
Ananthakrishnan et al. (53)	2013	1,200 patients for Crohn’s and UC	NLP techniques	Improving EMR case definitions for IBD	Achieved AUC of 0.95 for CD and 0.94 for UC.
Wang et al. (72)	2024	200 medical discharge summaries	GPT-4	Classifying GI bleeding events	GPT-4 showed high accuracy (94.4% for identifying GI bleeding), outperforming ICD codes significantly and demonstrating comparable or slightly lower accuracy to human reviewers
Hepatology					
Benedicenti et al. (56)	2023	56 gastroenterologists, 25 residents, 31 specialists	GPT-3	Answering clinical vignettes on Hepatology and Gastroenterology	Demonstrated improvement over time, underperformed vs. humans
Li et al. (24)	2023	1,463 postoperative colorectal cancer patients	NLP and machine learning integration	Predicting liver metastases	High accuracy in risk prediction
Wang et al. (41)	2022	LiverTox database	DeepCausality framework	Causal inference for drug-induced liver injury	Achieved 92% accuracy and an F1-score of 0.84 for DILI predictions.
Yeo et al. (29)	2023	164 questions about cirrhosis and hepatocellular carcinoma	GPT	Providing answers on cirrhosis and HCC	GPT provided accurate knowledge on cirrhosis (79.1% correct) and hepatocellular carcinoma (74% correct), although only a small proportion were considered comprehensive (cirrhosis 47.3%, HCC 41.1%).
Sherman et al. (17)	2024	3,134 patients with liver disease	NLP	Classifying liver disease pathology	The NLP model achieved high positive and negative predictive values (93.5–100%) across different histological features
Van Vleck et al. (30)	2019	38,575 patients	CLiX clinical NLP engine	Identifying NAFLD patients and disease progression	The NLP model demonstrated superior sensitivity and F2 scores compared to ICD codes and text searches. Sensitivity of 0.93 and an F2 score of 0.92 in identifying NAFLD
Sada et al. (71)	2016	1,138 patients identified from ICD-9 codes	Automated Retrieval Console (ARC)	Improving identification of hepatocellular cancer	Combining ICD-9 codes with NLP improved HCC identification: pathology (PPV 0.96, sensitivity 0.96, specificity 0.97), radiology (PPV 0.75, sensitivity 0.94, specificity 0.68)
Pradhan et al. (28)	2024	22 patients/caregivers and transplant hepatologists	Multiple LLMs	Generating patient educational materials about cirrhosis	AI materials matched human readability but were rated less actionable.
Schneider et al. (54)	2023	2.15 million pathology and 2.7 million imaging reports	Rule-based NLP algorithm	Identifying hepatic steatosis	Identified 3,007 biopsy-proven NAFLD cases and 42,083 imaging-proven cases, with a PPV of 99.7%.

AI, artificial intelligence; AUC, area under the curve; BARD, Google’s Generative AI Model; BioBERT, biomedical bidirectional encoder representations from transformers; cTAKES, clinical text analysis and knowledge extraction system; CD, Crohn’s disease; CDSS, clinical decision support system; CLAMP, clinical language annotation modeling and processing; CLiX, clinical information extraction; CRC, colorectal cancer; DILI, drug-induced liver injury; EMR, electronic medical record; ENDOSC, endoscopic submucosal dissection; EIM, extraintestinal manifestations; ERNIE, enhanced representation through knowledge integration; F1, F1-score; GI, gastrointestinal; GPT, generative pre-trained transformer; HCC, hepatocellular carcinoma; IBD, inflammatory Bowel disease; ICD, international classification of diseases; LSTM, long short-term memory; MMR, mismatch repair; NAFLD, non-alcoholic fatty liver disease; NASH, non-alcoholic steatohepatitis; NLP, natural language processing; NPV, negative predictive value; OCR, optical character recognition; PPV, positive predictive value; SPS, serrated polyposis syndrome; UC, ulcerative colitis.

**Table 2:** Included studies designs, comparisons and validations methods.

Author	Study design	Field + Specific interest	Model	Human comparator	Validation	Limitations
Gastroenterology						
Kong et al. (38)	Comparative analysis	Gastroenterology, *H. pylori*	ChatGPT 4.0, ChatGPT 3.5, ERNIE Bot 4.0	None	None	Limited completeness and possible bias in non-English settings.
Lahat et al. (46)	Cross-sectional analysis	Gastroenterology, General	GPT	3 Gastroenterologists	None	Variation in response quality; occasional inaccuracies.
Truhn et al. (49)	Retrospective analysis	Gastroenterology, CRC	GPT-4	Manual data extraction	Internal	Challenges with OCR accuracy; handling of handwritten notes.
Zhou et al. (48)	Retrospective analysis	Gastroenterology, Gastric cancer	GPT-3.5 and GPT-4	None	None	Importance of human oversight and detail accuracy limitations.
Choo et al. (39)	Prospective analysis	Gastroenterology, CRC	GPT	MDT decisions	None	Small sample size; retrospective nature.
Huo et al. (44)	Retrospective analysis	Gastroenterology, CRC screening	ChatGPT, Bing Chat, Google Bard, Claude 2	None	None	Variability in responses between chatbots; static data collection point.
Imler et al. (58)	Retrospective cohort study	Gastroenterology, Colonoscopy (adenoma detection)	cTAKES NLP engine	Manual review	Internal	Single institution study; template-driven reports.
Lim et al. (33)	Retrospective analysis	Gastroenterology, CRC screening	GPT-4	None	Internal	Free-text input limitations; lack of standardized prompts.
Imler et al. (36)	Retrospective analysis	Gastroenterology, Colonoscopy intervals	cTAKES NLP engine	Paired, blinded experts	None	Difficulty with complex cases needing manual review.
Bae et al. (60)	Retrospective analysis	Gastroenterology, Colonoscopy quality	Regular expressions, smartTA	5 Human annotators	Internal	Dataset-specific NLP system; reliance on accurate input data.
Denny et al. (63)	Retrospective analysis	Gastroenterology, CRC screening	KnowledgeMap Concept Identifier	Manual chart review	Internal	Single-center study with small sample size.
Lahat et al. (47)	Retrospective analysis	Gastroenterology, Research questions	GPT	3 Gastroenterologists	None	Small expert panel; subjective ratings.
Laique et al. (26)	Retrospective analysis	Gastroenterology, CRC screening	OCR + NLP	Manual review	Internal & External	Variability in documentation styles; reliance on high-quality scans.
Blumenthal et al. (65)	Retrospective analysis	Gastroenterology, Colonoscopy adherence	QPID NLP tool	None	Internal	Sample representativeness; generalizability to other settings.
Harkema et al. (42)	Retrospective analysis	Gastroenterology, Colonoscopy quality	Rule-based NLP engine	Manual annotations	Internal	Single institution study; mix of report styles.
Raju et al. (59)	Retrospective analysis	Gastroenterology, Colonoscopy ADR	Custom NLP software	Manual review	None	Institution-specific study; not tested on other systems.
Nayor et al. (57)	Retrospective analysis	Gastroenterology, Colonoscopy ADR	Custom NLP pipeline	Manual review	Internal	Misclassification risks; complexity of free text.
Atarere et al. (25)	Cross-sectional analysis	Gastroenterology, CRC screening	ChatGPT, BingChat, YouChat™	Board-certified physicians	None	Models not designed for medical use; reliance on training data.
Seong et al. (40)	Retrospective analysis	Gastroenterology, Colonoscopy	Bi-LSTM-CRF	None	Internal	Single institution study; reporting style variations.
Lee et al. (21)	Cross-sectional analysis	Gastroenterology, Colonoscopy quality	Commercial NLP tool	Manual chart review	Internal	Single healthcare system study; dependency on physician reports.
Denny et al. (50)	Retrospective analysis	Gastroenterology, CRC screening	KnowledgeMap Concept Identifier	Manual review	Internal	Focus on colonoscopies; error sources in date references.
Parthasarathy et al. (18)	Retrospective cohort study	Gastroenterology, CRC screening	NLP	Clinicians	None	7% error rate in data extraction.
Rammohan et al. (68)	Prospective analysis	Gastroenterology, General	GPT-4, Bard	None	None	Focus on specific questions; limited to two AI tools.
Pereyra et al. (37)	Prospective observational study	Gastroenterology, CRC	GPT-3.5	Physicians	None	Small number of vignettes; outdated model.
Song et al. (61)	Retrospective analysis	Gastroenterology, Gastroscopy	Custom NLP pipeline	Gastroenterologists	Internal	Specificity to data formatting; need for manual updates.
Peng et al. (45)	Prospective observational study	Gastroenterology, CRC	GPT-3.5	Expert answers	Internal	Limited question scope from a reference book.
Tinmouth et al. (70)	Retrospective analysis	Gastroenterology, Colonoscopy ADR	NLP	Expert review	Internal	Voluntary reporting system; procedure-specimen mismatch.
Mehrotra et al. (27)	Cross-sectional analysis	Gastroenterology, Colonoscopy	NLP (C-QUAL)	Physician manual review	Internal	Single healthcare system study.
Becker et al. (64)	Retrospective analysis	Gastroenterology, CRC	German-specific NLP	Manual review	Internal	Documentation complexity; moderate performance in some areas.
Hou et al. (31)	Retrospective analysis	Gastroenterology, IBD surveillance	ARC	Gastroenterologist	Internal	Pathology reporting variability.
Gorelik et al. (51)	Prospective observational study	Gastroenterology, Postcolonoscopy	GPT-4	Society guidelines	None	Inherent randomness and outdated training data.
Samaan et al. (34)	Cross-sectional analysis	Gastroenterology, Cirrhosis	GPT	Transplant hepatologist	None	Model hallucinations; Arabic response accuracy gap.
Cankurtaran et al. (67)	Retrospective analysis	Gastroenterology, IBD	GPT-4	None	None	Response variability; lack of detail in treatment advice.
Wenker et al. (69)	Retrospective analysis	Gastroenterology, Barrett’s Esophagus	CLAMP	Manual review	Internal & External	VA sample limits generalizability.
Imler et al. (66)	Retrospective cohort study	Gastroenterology, ERCP	Apache UIMA-based NLP	Gastroenterologist	Internal	Single-center bias; ICD coding assumptions.
Li et al. (62)	Retrospective analysis	Gastroenterology and hepatology	ML and NLP fusion	Two experienced physicians	External	Scale of data integration and complexity; limited interpretability.
Li et al. (16)	Retrospective cohort study	Gastroenterology, Upper GI cancer	ENDOANGEL-AS	Physicians	Internal & External	Annotation variability; semi-structured data limits.
Wagholikar et al. (35)	Retrospective analysis	Gastroenterology, Colonoscopy	NLP	Gastroenterologist	Internal	Single expert; single institution.
Sciberras et al. (20)	Prospective study	Gastroenterology, IBD	GPT-3.5	None	None	Lacks detailed responses; occasional inaccuracies.
Stidham et al. (52)	Retrospective cohort study	Gastroenterology, IBD	NLP	Human reviewers	Internal	Source document variation limits generalizability.
Ganguly et al. (22)	Retrospective analysis	Gastroenterology, Colonoscopy ADR	NLP	Manual review	Internal	Human input reliance; data integration complexity.
Ma et al. (43)	Retrospective analysis	Gastroenterology, Esophageal ESD	GPT-3.5	Human operators	Internal	Single-center dataset limits; small prompt optimization cases.
Gravina et al. (32)	Cross-sectional analysis	Gastroenterology, Education	GPT 3.5, Perplexity AI	None	Internal	AI education lacks oversight; variable performance.
Fevrier et al. (84)	Retrospective cohort study	Gastroenterology, Colonoscopy	NLP tool	Manual review	Internal	Incomplete documentation and specimen data challenges.
Benson et al. (55)	Retrospective analysis	Gastroenterology, Colonoscopy	NLP	Manual annotations	None	Report structure adaptations; evaluation of rare features limited.
Zand et al. (23)	Retrospective cohort study	Gastroenterology, IBD	NLP	3 Physicians	None	Homogeneous patient sample limits generalizability.
Ananthakrishnan et al. (53)	Retrospective analysis	Gastroenterology, IBD	NLP	None	Internal	Single healthcare system; needs broader validation.
Wang et al. (72)	Retrospective analysis	Gastroenterology, GI bleeding	GPT-4	Human reviewers	None	Single clinical scenario focus; model specificity.
Hepatology						
Benedicenti et al. (56)	Cross-sectional analysis	Gastroenterology, Education	GPT-3	Gastroenterologists	None	Performance variability; static format.
Li et al. (24)	Retrospective cohort study	Hepatology, HCC	NLP	Manual review	External	Limited generalizability; physician agreement variability.
Wang et al. (41)	Retrospective analysis	Hepatology, DILI	DeepCausality	None	Internal	Dependency on structured data; specificity to LiverTox.
Yeo et al. (29)	Retrospective analysis	Hepatology, Cirrhosis	GPT	Transplant hepatologists	None	Inconsistent comprehensiveness; limited regional guideline knowledge.
Sherman et al. (17)	Retrospective cohort study	Hepatology, MASLD	NLP	Manual review	Internal	Heterogeneous biopsy data; evolving MASLD definitions.
Van Vleck et al. (30)	Retrospective cohort study	Hepatology, NAFLD	CLiX NLP engine	Manual validation	Internal	Generalizability limits; dependency on physician impressions.
Sada et al. (71)	Retrospective analysis	Hepatology, HCC	ARC	Manual classification	Internal and External	VA-specific data limits generalizability.
Pradhan et al. (28)	Retrospective analysis	Hepatology, Cirrhosis	Multiple LLMs	Human materials	None	AI not designed for medical use; lack of visual aids.
Schneider et al. (54)	Retrospective analysis	Hepatology, NAFLD	NLP	Manual review	Internal	Bias in EHR analysis; NLP interpretation challenges.

AI, artificial intelligence; ADR, adenoma detection rate; ARC, automated retrieval console; CDSS, clinical decision support system; CRC, colorectal cancer; cTAKES, clinical text analysis and knowledge extraction system; DILI, drug-induced liver injury; EHR, electronic health records; EMR, electronic medical records; ESD, endoscopic submucosal dissection; GI, gastrointestinal; GPT, generative pre-trained transformer; HCC, hepatocellular carcinoma; IBD, inflammatory Bowel disease; IHC, immunohistochemistry; LLM, large language model; MDT, multidisciplinary tumor board; ML, machine learning; NAR, non-adherence ratio; NASH, non-alcoholic steatohepatitis; NAFLD, non-alcoholic fatty liver disease; NLP, natural language processing; NPV, negative predictive value; OCR, optical character recognition; PPV, positive predictive value; SPS, serrated polyposis syndrome; USMSTF, U.S. multi-society task force.

### Disease detection and diagnosis

**Figure 4:**
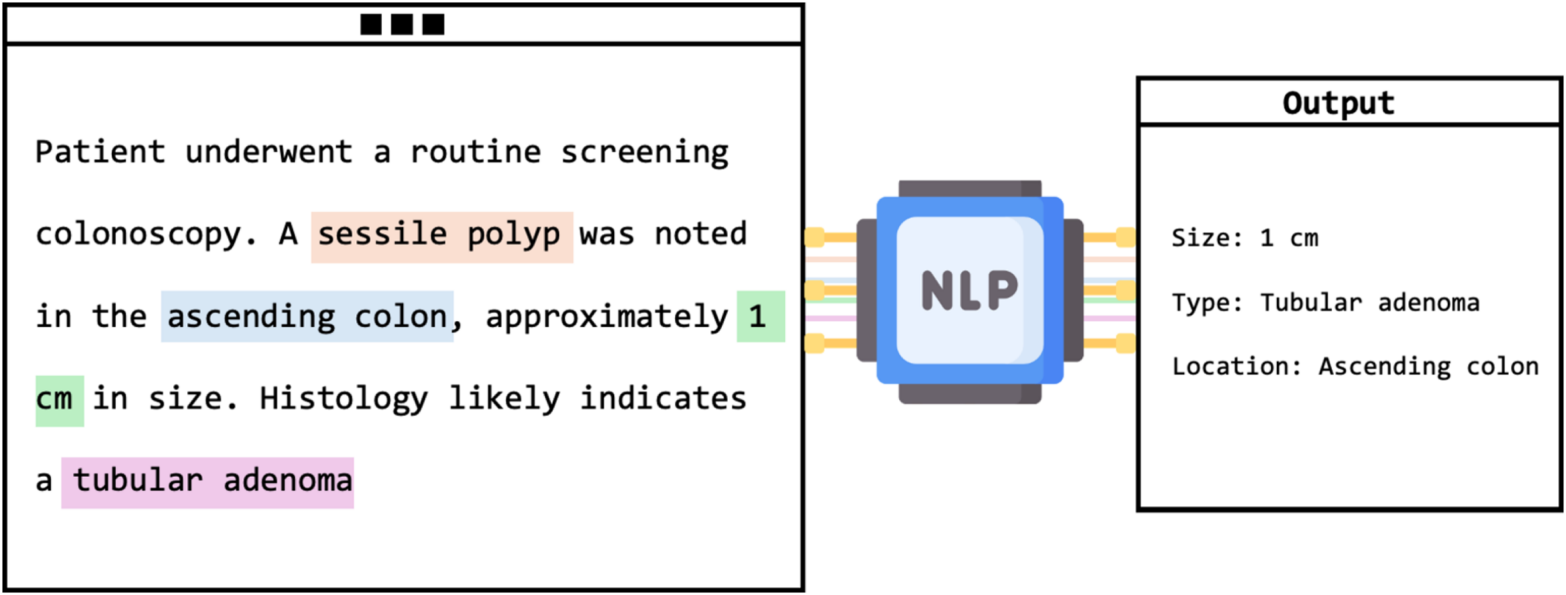
Visual framework of NLP extracting adenoma characteristics from unstructured colonoscopy report.

Most of the studies evaluated NLP models in extracting data from colonoscopy reports (*n* = 17) (Figure 4). Nonetheless, there were many unique applications.

#### Colonoscopy reports

This category, which includes 17 studies, primarily explored NLP’s role in enhancing the interpretation of unstructured colonoscopy reports. Various quality and diagnostic measures were evaluated, such as the adenoma detection rate (ADR), a frequent subject of investigation. For instance, Nayor et al. reported that their NLP pipeline achieved high precision and recall in the automated calculation of ADR (57). Other assessments included polyp detection and sizing, with Imler et al. demonstrating accuracies of 98% for pathology level identification and 96% for size estimation (58). Additionally, Raju et al. noted that NLP matched or exceeded manual methods in identifying and categorizing adenomas with a detection rate of 43% (59). Overall, NLP models showed a broad range of accuracies from 84 to 100%, consistently outperforming manual review methods. Despite needing GPUs, these models reduce the time and effort of manual evaluations.

#### Other diagnostic applications

Beyond colonoscopy, NLP was applied to a diverse array of diagnostic contexts in gastroenterology and hepatology.

In gastroenterology, several innovative NLP applications have emerged. For example, Wenker et al. utilized NLP to identify dysplasia in Barrett’s Esophagus from esophagogastroduodenoscopy (EGD) reports with a high accuracy of 98.7% (69). Song et al. developed a model to extract detailed clinical information such as disease presence, location, size, and stage from unstructured EGD reports, achieving high sensitivity, precision, and accuracy scores (61). Denny et al. applied NLP to enhance colorectal cancer screening by identifying references to four CRC tests within electronic clinical documentation, demonstrating superior recall compared to traditional manual and billing record reviews (63). Additionally, Blumenthal et al. and Parthasarathy et al. used NLP for patient monitoring, with the former detecting non-adherence to follow-up colonoscopies with an AUC of 70.2%, and the latter identifying patients meeting WHO criteria for serrated polyposis syndrome with 93% accuracy (18, 65).

For IBDs, Stidham et al. utilized NLP to detect and infer the activity status of extraintestinal manifestations from clinical notes, enhancing detection accuracy to 94.1% and specificity to 95% (52). Ananthakrishnan et al. explored improving case definitions for Crohn’s disease and ulcerative colitis by combining codified data with narrative clinical texts, which identified 6–12% more patients than models using codified data alone, with AUCs of 0.95 for Crohn’s disease and 0.94 for ulcerative colitis (53).

In hepatology, NLP has facilitated significant advancements in disease identification and progression monitoring. Sada et al. combined NLP with ICD-9 codes to improve the identification of hepatocellular carcinoma cases from EHR data, significantly enhancing sensitivity and specificity, with an F2 score of 0.92 (71). Van Vleck et al. employed NLP to track disease progression in patients with non-alcoholic fatty liver disease (NAFLD), demonstrating superior sensitivity and F2 scores compared to traditional methods, effectively identifying disease progression from NAFLD to NASH or cirrhosis with sensitivity of 0.93 and an F2 score of 0.92 (30). Furthermore, Sherman et al. developed an NLP model capable of automatically scoring and classifying histological features found in pathology reports related to metabolic associated steatohepatitis (17). The goal was to estimate the risk of progression towards cirrhosis. The model demonstrated high positive and negative predictive values, ranging from 93.5 to 100%, across various histological features (17). Importantly, this NLP model facilitated the creation of a large and quality-controlled cohort of MASLD patients (17).

### Patient care

The patient care section is subdivided into two categories: patient management and communication, which comprises 13 studies, and clinical decision support, encompassing 9 studies.

#### Management and communication

This category explores the use of NLP and LLMs in facilitating communication and management.

In gastroenterology, studies like Lahat et al. evaluated ChatGPT’s ability to answer real-life gastroenterology-related patient queries, achieving moderate effectiveness with accuracy scores ranging from 3.4 to 3.9 (47). Choo et al. reported an 86.7% concordance rate between ChatGPT’s recommendations for managing complex colorectal cancer cases and decisions made by multidisciplinary teams (39). Furthermore, Lim et al. demonstrated that a contextualized GPT-4 model provided accurate colonoscopy interval advice, significantly outperforming standard models by adhering closely to established guidelines (33). Imler et al. used the cTAKES system to achieve an 81.7% agreement with guideline-adherent colonoscopy surveillance intervals, substantially surpassing manual review accuracies (36). However, studies like Huo et al. and Atarere et al. indicated variability in ChatGPT’s performance, suggesting the need for enhancements in AI consistency and reliability (25, 44). In the area of IBD, Zand et al. developed an NLP model that categorized electronic dialog data, showing a 95% agreement with physician evaluations and underscoring the potential of automated chatbots in patient interaction (23). Sciberras et al. found ChatGPT to provide highly accurate (84.2%) and moderately complete responses to patient inquiries about IBD, with particular strengths in topics like smoking and medication (20).

In hepatology, Yeo et al. tested GPT’s proficiency in delivering emotional support and accurate information on cirrhosis and hepatocellular carcinoma, achieving correct response rates of 79.1% for cirrhosis and 74% for carcinoma (29). Samaan et al. explored GPT’s effectiveness in Arabic, noting a 72.5% accuracy rate, though it was less accurate than its English counterpart, indicating disparities in language performance (34).

#### Clinical decision support

NLP models were tested for their accuracy and effectiveness in decision-making scenarios. For example, Kong et al. evaluated LLMs’ capability to provide counseling on *Helicobacter pylori*, noting that while accuracy was generally high (90% acceptable responses), completeness needed improvement (38). Li et al.’s integration of NLP with machine learning for predicting liver metastases showed impressive results with accuracy and F1 scores around 80.4% (24). The study by Becker et al. utilized an NLP pipeline tailored for German, achieving high precision and recall in guideline-based treatment extraction from clinical notes (64). Further, Wang et al.’s “DeepCausality” framework accurately assessed causal factors for drug-induced liver injuries, aligning well with clinical guidelines (41). Another significant study, Wagholikar et al., demonstrated that an NLP-powered clinical decision support system could assist in making guideline-adherent recommendations for colonoscopy surveillance, as it made optimal recommendations in 48 out of 53 cases (35).

### Education and research

Five studies focused on this aspect. Generally, NLP and LLMs have demonstrated a promising capacity to enhance learning and knowledge dissemination. Benedicenti et al. explored the accuracy of ChatGPT in solving clinical vignettes against gastroenterologists, noting an initial 40% accuracy that improved to 65% over time, suggesting a potential for future clinical integration with continued advancements (56). Zhou et al. assessed GPT-3.5 and GPT-4 for their ability to provide consultation recommendations and analyze gastroscopy reports related to gastric cancer, with GPT-4 achieving 91.3% appropriateness and 95.7% consistency (48). Lahat et al. utilized GPT to generate research questions in gastroenterology, finding the questions relevant and clear but lacking in originality (46). Meanwhile, Gravina et al. highlighted the efficacy of ChatGPT 3.5 in medical education, as it outperformed Perplexity AI in residency exam questions with a 94.11% accuracy rate (32). Additionally, Pradhan et al. compared AI-generated patient educational materials on cirrhosis with human-derived content, finding no significant differences in readability or accuracy, though human materials were deemed more actionable (28).

### Validation and comparisons

Of the 57 studies, only 5 performed external validation using independent datasets. A total of 30 studies used internal validation, with 27 applying classical subsets of the same data for re-testing and validating their main results. Three studies employed a method of running LLM prompts multiple times (2–3 times) to assess the consistency of responses. Meanwhile, 22 studies did not perform any validation. Regarding direct comparisons of NLPs and LLMs with human counterparts, 44 studies compared the model’s performance with manual review by physicians or manual data extraction methods. The number of human reviewers varied between studies, ranging from 1 to 5. Thirteen studies did not perform direct comparisons (Table 2).

## Discussion

Our systematic review assessed the integration of NLP and LLMS in gastroenterology and hepatology, registering significant advancements. We reviewed 57 studies, highlighting a sharp increase in research over the last 2 years, particularly focusing on newer models like GPT-3 and GPT-4. These studies reflect a shift from traditional tasks, such as report analysis, to more dynamic roles in patient management and research facilitation.

To present the findings in a clear and easily interpretable manner, we opted to categorize the reviewed studies into a minimal set of broad application areas. We acknowledge that these categories are not absolute and that certain studies may naturally span multiple domains (for example, colonoscopy surveillance intervals, while placed under one heading, could also be considered a form of clinical decision support). Nonetheless, by grouping the research into broader, more encompassing categories, we aimed to give readers a high-level understanding of where progress is most pronounced, and which areas appear closer to real-world clinical integration.

The results show that certain NLP applications seem ready for immediate clinical use. For example, Schneider et al. (2023) identified 42,000 hepatic steatosis cases using an NLP model on 2.15 million pathology reports and 2.7 million imaging reports. This level of precision (PPV 99.7%) exemplifies NLP’s readiness to support diagnostic processes in large-scale healthcare settings. Similarly, Truhn et al. (2024) successfully employed GPT-4 to extract structured data from colorectal cancer reports with a precision of 99% for T-stage identification, suggesting a high reliability of NLP in processing and structuring complex pathological data.

Conversely, the technology’s expansion into more dynamic roles such as comprehensive disease management and holistic patient care is still evolving. For instance, Kong et al. (2024) found that while the accuracy and comprehensibility of GPT-4’s responses to medical inquiries about *Helicobacter pylori* were high, the completeness of the information was less satisfactory. This indicates ongoing challenges in ensuring that NLP outputs are not only accurate but also fully informative.

Our results suggest that both classic NLP methods and newer models can be effectively integrated to streamline manual tasks such as extracting data and making diagnoses from complex and unstructured reports, with an accuracy that typically surpasses manual screening (16–18, 21, 22, 27, 33). This builds upon and adds on a previous systematic review of NLP in gastroenterology and hepatology conducted by Hou et al. (2). While he found promising results, he emphasized the need for careful consideration of the quality of clinical data within EHRs, and also highlighted the importance of understanding variations and deviations from established clinical practice standards (2). Our updated results indicate that these models consistently demonstrate high accuracies (16–18, 21, 22, 27, 33). This trend is observable in other fields utilizing NLP, such as radiology and infectious diseases (74, 75). However, our research suggests that applying these methods to more complex tasks like patient management, education, and clinical decision-making is still challenging (20, 29, 34, 37). While newer models show promising results, there are significant limitations and variability that require further development (67). This trend is consistent with data and the current findings from other fields (76, 77).

Several limitations of our review must be acknowledged. Many studies utilize single-institution datasets, which could affect the generalizability of the findings. This is important especially because only 5 studies (8.7%) reported performing an external validation. The accuracy of NLP outputs is heavily dependent on the quality of the input data, with errors or inconsistencies in medical records potentially leading to inaccurate results (78). The opaque nature of AI decision-making processes (‘black box’) raises concerns about the transparency and trustworthiness of these models in clinical settings (79). Ethical considerations around potential biases in training data and algorithmic outputs underscore the necessity for careful implementation to ensure fairness and equity in healthcare delivery (80). Moreover, the accuracy and reliability of NLP and LLM outputs are directly tied to the quality of the input data. EHRs, clinical notes, and imaging reports often contain incomplete, ambiguous, or inaccurately recorded information. These data imperfections can lead to propagation of errors and compound biases within the model’s output, potentially influencing clinical decision-making and patient care. Additionally, while many NLP and LLM models show promise in structured tasks like disease detection or data extraction, they remain susceptible to “hallucinations”—generating plausible-sounding but factually incorrect statements (81). Such errors, if undetected, may result in misguided clinical judgments, suboptimal patient management, and delayed interventions. An additional critical dimension of these limitations involves the potential for algorithmic biases, including those related to sociodemographic factors such as race, ethnicity, gender, language proficiency, and socioeconomic status (82, 83). Models trained on unrepresentative or historically biased data risk perpetuating systemic inequalities in healthcare. Despite the promising accuracy of some NLP applications, they are not yet widely integrated into day-to-day clinical workflows, particularly for patient care and decision-making; current limitations and the need for thorough testing and validation—especially for newer, less researched techniques—have thus far hindered their routine implementation in practice.

In conclusion, our systematic review highlights the impact of NLP and LLMs in gastroenterology and hepatology. On one hand, NLP has already proven its utility in screening and analyzing medical reports, facilitating streamlined screening policies with impressive outcomes. On the other hand, the capabilities of newer LLMs are still unfolding, with their full potential in complex management and research roles yet to be fully realized. The results demonstrate that while some applications of NLP are well-established and highly effective, newer LLMs offer exciting, emerging applications that promise to further enhance clinical practice. Moving forward, research focus should be on refining these models, and externally validating the results to ensure prospectively they meet real-world clinical needs.

## Data Availability

The original contributions presented in the study are included in the article/Supplementary material, further inquiries can be directed to the corresponding author.
