# Supplementary material for: Emerging applications of NLP and large language models in gastroenterology and hepatology: a systematic review
Source: Front Med (Lausanne). 2025 Jan 22;11:1512824. doi: 10.3389/fmed.2024.1512824 (PMC11799763; doi:10.3389/fmed.2024.1512824)
Supplement: Supplementary file 1 [file Data_Sheet_1.PDF]

Supplementary Materials

Advances in Gastroenterology and Hepatology: A Systematic Review of NLP and LLM Applications.

Table of contents

- Specific Booleans used in each database --- 2
  - PubMed --- 2
  - Embase --- 5
  - Scopus --- 5
  - Web of science --- 5
  - Cochrane library – 6
  - IEEE Xplore – 6
- Risk of Bias tables --- 7
  - PROBAST --- 7
  - QUADAS --- 7
  - ROBINS – I --- 9
- Journals, quartiles and SJR's of the included studies --- 11

Specific Booleans used in each database.

PubMed

Search number	Query	Filters	Search Details	Results
**5**	("natural language processing" OR "NLP" OR "large language models" OR "LLM" OR "BERT" OR "GPT-3" OR "GPT" OR "ChatGPT" OR "GPT-3.5" OR "GPT-4" OR "transformer language models") AND ("gastroenterology" OR "digestive health" OR "gastrointestinal oncology" OR "endoscopic imaging" OR "colorectal cancer screening" OR "hepatocellular carcinoma" OR "liver fibrosis" OR "inflammatory bowel disease management" OR "IBD" OR "Crohn's disease" OR "ulcerative colitis treatment")	English, from 2003 - 2024	(("natural language processing"[All Fields] OR "NLP"[All Fields] OR "large language models"[All Fields] OR "LLM"[All Fields] OR "BERT"[All Fields] OR "GPT-3"[All Fields] OR "GPT"[All Fields] OR "ChatGPT"[All Fields] OR "GPT-3.5"[All Fields] OR "GPT-4"[All Fields] OR "transformer language models"[All Fields]) AND ("gastroenterology"[All Fields] OR "digestive health"[All Fields] OR "gastrointestinal oncology"[All Fields] OR "endoscopic imaging"[All Fields] OR "colorectal cancer screening"[All Fields] OR "hepatocellular carcinoma"[All Fields] OR "liver fibrosis"[All Fields] OR "inflammatory bowel disease management"[All Fields] OR "IBD"[All Fields] OR "Crohn's disease"[All Fields] OR "ulcerative colitis treatment"[All Fields])) AND ((english[Filter]) AND (2003:2024[pdat]))	393
**4**	("natural language processing" OR "NLP" OR "large language models" OR "LLM" OR "BERT" OR "GPT-3" OR "GPT" OR "ChatGPT" OR "GPT-3.5" OR "GPT-4" OR "transformer language models") AND ("gastroenterology" OR "digestive health" OR "gastrointestinal oncology" OR "endoscopic imaging" OR "colorectal cancer screening" OR "hepatocellular carcinoma" OR "liver fibrosis" OR "inflammatory bowel disease management" OR "IBD" OR	from 2003 - 2024	(("natural language processing"[All Fields] OR "NLP"[All Fields] OR "large language models"[All Fields] OR "LLM"[All Fields] OR "BERT"[All Fields] OR "GPT-3"[All Fields] OR "GPT"[All Fields] OR "ChatGPT"[All Fields] OR "GPT-3.5"[All Fields] OR "GPT-4"[All Fields] OR "transformer language models"[All Fields]) AND ("gastroenterology"[All Fields] OR "digestive health"[All Fields] OR "gastrointestinal oncology"[All Fields] OR "endoscopic imaging"[All Fields] OR "colorectal cancer screening"[All Fields] OR "hepatocellular carcinoma"[All Fields] OR "liver fibrosis"[All Fields] OR "inflammatory bowel disease management"[All Fields] OR "IBD"[All Fields] OR "Crohn's disease"[All	404

	"Crohn's disease" OR "ulcerative colitis treatment")		Fields] OR "ulcerative colitis treatment"[All Fields])) AND (2003:2024[pdat])	
**3**	("natural language processing" OR "NLP" OR "large language models" OR "LLM" OR "BERT" OR "GPT-3" OR "GPT" OR "ChatGPT" OR "GPT-3.5" OR "GPT-4" OR "transformer language models") AND ("gastroenterology" OR "digestive health" OR "gastrointestinal oncology" OR "endoscopic imaging" OR "colorectal cancer screening" OR "hepatocellular carcinoma" OR "liver fibrosis" OR "inflammatory bowel disease management" OR "IBD" OR "Crohn's disease" OR "ulcerative colitis treatment")		("natural language processing"[All Fields] OR "NLP"[All Fields] OR "large language models"[All Fields] OR "LLM"[All Fields] OR "BERT"[All Fields] OR "GPT-3"[All Fields] OR "GPT"[All Fields] OR "ChatGPT"[All Fields] OR "GPT-3.5"[All Fields] OR "GPT-4"[All Fields] OR "transformer language models"[All Fields]) AND ("gastroenterology"[All Fields] OR "digestive health"[All Fields] OR "gastrointestinal oncology"[All Fields] OR "endoscopic imaging"[All Fields] OR "colorectal cancer screening"[All Fields] OR "hepatocellular carcinoma"[All Fields] OR "liver fibrosis"[All Fields] OR "inflammatory bowel disease management"[All Fields] OR "IBD"[All Fields] OR "Crohn's disease"[All Fields] OR "ulcerative colitis treatment"[All Fields])	474
**2**	(("natural language processing" OR "NLP" OR "large language models" OR "LLM" OR "BERT" OR "GPT-3" OR "GPT" OR "ChatGPT" OR "GPT-3.5" OR "GPT-4" OR "transformer language models" OR "deep learning" OR "machine learning" OR "AI" OR "artificial intelligence" OR "text mining" OR "computational linguistics") AND ("gastroenterology" OR "digestive health" OR "gastrointestinal oncology" OR "endoscopic imaging" OR "colorectal cancer screening" OR "hepatocellular carcinoma" OR "liver fibrosis" OR "liver diseases" OR "gastrointestinal diseases" OR "digestive system diseases" OR "inflammatory bowel disease	from 2003 - 2024	(("natural language processing"[All Fields] OR "NLP"[All Fields] OR "large language models"[All Fields] OR "LLM"[All Fields] OR "BERT"[All Fields] OR "GPT-3"[All Fields] OR "GPT"[All Fields] OR "ChatGPT"[All Fields] OR "GPT-3.5"[All Fields] OR "GPT-4"[All Fields] OR "transformer language models"[All Fields] OR "deep learning"[All Fields] OR "machine learning"[All Fields] OR "AI"[All Fields] OR "artificial intelligence"[All Fields] OR "text mining"[All Fields] OR "computational linguistics"[All Fields]) AND ("gastroenterology"[All Fields] OR "digestive health"[All Fields] OR "gastrointestinal oncology"[All Fields] OR "endoscopic imaging"[All Fields] OR "colorectal cancer screening"[All Fields] OR "hepatocellular carcinoma"[All Fields] OR "liver fibrosis"[All Fields] OR "liver diseases"[All Fields] OR "gastrointestinal diseases"[All Fields] OR "digestive system diseases"[All Fields] OR "inflammatory bowel disease management"[All Fields] OR "IBD"[All Fields] OR "Crohn's disease"[All Fields] OR "ulcerative colitis	13,056

	management" OR "IBD" OR "Crohn's disease" OR "ulcerative colitis treatment" OR "gastrointestinal inflammation" OR "gastrointestinal imaging" OR "gastrointestinal diagnostics"))		treatment"[All Fields] OR "gastrointestinal inflammation"[All Fields] OR "gastrointestinal imaging"[All Fields] OR "gastrointestinal diagnostics"[All Fields])) AND (2003:2024[pdat])	
**1**	(("natural language processing" OR "NLP" OR "large language models" OR "LLM" OR "BERT" OR "GPT-3" OR "GPT" OR "ChatGPT" OR "GPT-3.5" OR "GPT-4" OR "transformer language models" OR "deep learning" OR "machine learning" OR "AI" OR "artificial intelligence" OR "text mining" OR "computational linguistics") AND ("gastroenterology" OR "digestive health" OR "gastrointestinal oncology" OR "endoscopic imaging" OR "colorectal cancer screening" OR "hepatocellular carcinoma" OR "liver fibrosis" OR "liver diseases" OR "gastrointestinal diseases" OR "digestive system diseases" OR "inflammatory bowel disease management" OR "IBD" OR "Crohn's disease" OR "ulcerative colitis treatment" OR "gastrointestinal inflammation" OR "gastrointestinal imaging" OR "gastrointestinal diagnostics"))		("natural language processing"[All Fields] OR "NLP"[All Fields] OR "large language models"[All Fields] OR "LLM"[All Fields] OR "BERT"[All Fields] OR "GPT-3"[All Fields] OR "GPT"[All Fields] OR "ChatGPT"[All Fields] OR "GPT-3.5"[All Fields] OR "GPT-4"[All Fields] OR "transformer language models"[All Fields] OR "deep learning"[All Fields] OR "machine learning"[All Fields] OR "AI"[All Fields] OR "artificial intelligence"[All Fields] OR "text mining"[All Fields] OR "computational linguistics"[All Fields]) AND ("gastroenterology"[All Fields] OR "digestive health"[All Fields] OR "gastrointestinal oncology"[All Fields] OR "endoscopic imaging"[All Fields] OR "colorectal cancer screening"[All Fields] OR "hepatocellular carcinoma"[All Fields] OR "liver fibrosis"[All Fields] OR "liver diseases"[All Fields] OR "gastrointestinal diseases"[All Fields] OR "digestive system diseases"[All Fields] OR "inflammatory bowel disease management"[All Fields] OR "IBD"[All Fields] OR "Crohn's disease"[All Fields] OR "ulcerative colitis treatment"[All Fields] OR "gastrointestinal inflammation"[All Fields] OR "gastrointestinal imaging"[All Fields] OR "gastrointestinal diagnostics"[All Fields])	14,478

Embase

('natural language processing' OR 'nlp' OR 'bert' OR 'gpt-3' OR 'gpt' OR 'chatgpt' OR 'gpt-3.5' OR 'gpt-4' OR 'transformer language models' OR 'ai language technology') AND ('gastroenterology' OR 'hepatology' OR 'gastrointestinal oncology' OR 'endoscopic imaging' OR 'colorectal cancer screening' OR 'hepatocellular carcinoma' OR 'liver fibrosis' OR 'inflammatory bowel disease management' OR 'ibd' OR 'crohns disease' OR 'ulcerative colitis treatment')

AND (2003:py OR 2004:py OR 2005:py OR 2006:py OR 2007:py OR 2008:py OR 2009:py OR 2010:py OR 2011:py OR 2012:py OR 2013:py OR 2014:py OR 2015:py OR 2016:py OR 2017:py OR 2018:py OR 2019:py OR 2020:py OR 2021:py OR 2022:py OR 2023:py OR 2024:py) AND [embase]/lim NOT ([embase]/lim AND [medline]/lim) AND ('article'/it OR 'conference paper'/it)

Scopus

(TITLE-ABS-KEY(("natural language processing" OR "NLP" OR "large language models" OR "LLM" OR "BERT" OR "GPT-3" OR "GPT" OR "ChatGPT" OR "GPT-3.5" OR "GPT-4" OR "transformer language models") AND ("Gastroenterology" OR "Hepatology" OR "peptic ulcer disease" OR "PUD" OR "Endoscopy" OR "colorectal cancer" OR "hepatocellular carcinoma treatment" OR "IBD predictive modeling" OR "endoscopic imaging"))) AND PUBYEAR > 2002 AND (NOT DOCTYPE(re) OR DOCTYPE(le) OR DOCTYPE(ed) OR DOCTYPE(cr) OR DOCTYPE(cp))

Web of science

TS=("natural language processing" OR "NLP" OR "large language models" OR) "LLM" OR "BERT" OR "GPT-3" OR "GPT" OR "ChatGPT" OR "GPT-3.5" OR "GPT-4" OR "transformer language models") AND TS=("Gastroenterology" OR "Hepatology" OR "peptic ulcer disease" OR "PUD" OR "Endoscopy" OR "colorectal cancer" OR "hepatocellular carcinoma treatment" OR "IBD predictive modeling" OR "endoscopic imaging")) AND PY=(2003-2023) NOT DT=("Review" ("OR "Editorial Material" OR "Letter" OR "Meeting Abstract" OR "News Item")

Cochrane library

("natural language processing" OR "NLP" OR "large language models" OR "LLM" OR "BERT" OR "GPT-3" OR "GPT" OR "ChatGPT" OR "GPT-3.5" OR "GPT-4" OR "transformer language models") AND ("gastroenterology" OR "hepatology" OR "digestive health" OR "gastrointestinal oncology" OR "endoscopic imaging" OR "colorectal cancer screening" OR "hepatocellular carcinoma" OR "liver fibrosis" OR "inflammatory bowel disease management" OR "IBD" OR "Crohn's disease" OR "ulcerative colitis treatment")

IEEE Xplore

("natural language processing" OR "NLP" OR "large language models" OR "LLM" OR "BERT" OR "GPT-3" OR "GPT" OR "ChatGPT" OR "GPT-3.5" OR "GPT-4" OR "transformer language models") AND ("gastroenterology" OR "hepatology" OR "digestive health" OR "gastrointestinal oncology" OR "endoscopic imaging" OR "colorectal cancer screening" OR "hepatocellular carcinoma" OR "liver fibrosis" OR "inflammatory bowel disease management" OR "IBD" OR "Crohn's disease" OR "ulcerative colitis treatment")

Risk of Bias tables.

Additional information, such as sample sizes, years of publication and settings for each of the included papers can be found organized in the summary of the included studies table in the main manuscript.

Table S1: PROBAST.

	Risk of bias				Applicability		
**Author**	Participants	Predictors	Outcome	Analysis	Participants	Predictors	Outcome
**Li et al.**	low	low	low	low	low	low	low
**Blumenthal et al.**	low	low	low	low	high	low	low
**Wang et al. (2022)**	low	low	low	low	low	low	low
**Sherman et al.**	low	low	low	low	high	low	low

Tables S2: QUADAS.

	Risk of Bias				Applicability Concerns		
**Author**	Patient Selection	Index Test	Reference Standard	Flow and Timing	Patient Selection	Index Test	Reference Standard
**Schneider et al.**	low	low	low	unclear	low	low	low
**Truhn et al.**	low	low	low	low	low	low	low
**Imler et al. (2013)**	low	low	low	low	low	low	low
**Imler et al. (2014)**	low	low	low	low	high	low	low
**Bae et al.**	low	low	low	unclear	high	low	low
**Denny et al. (2012)**	low	low	low	low	high	low	low
**Laique et al.**	low	low	low	low	low	low	low
**Harkema et al.**	low	low	low	low	high	low	low
**Raju et al.**	low	low	low	unclear	low	low	low
**Nayor et al.**	low	low	low	low	low	low	low
**Seong et al.**	low	low	high	low	low	low	high

**Lee et al.**	low	low	low	unclear	high	low	low
**Denny et al. (2010)**	low	low	low	low	high	low	low
**Parthasarathy et al.**	low	low	low	low	low	low	low
**Song et al.**	low	low	low	unclear	high	low	low
**Tinmouth et al.**	high	low	low	low	high	low	low
**Mehrotra et al.**	low	low	low	low	low	low	low
**Becker et al.**	high	low	low	unclear	high	low	low
**Hou et al.**	low	low	low	low	high	low	low
**Wenker et al.**	low	low	low	unclear	low	low	low
**Imler et al. (2018)**	low	low	low	low	low	low	low
**Li et al. (2021)**	low	low	low	low	high	low	low
**Li et al. (2022)**	low	low	low	low	low	low	low
**Stidham et al.**	low	low	low	unclear	high	low	low
**Ganguly et al.**	low	low	low	unclear	low	low	low
**Van Vleck et al.**	low	low	low	low	low	low	low
**Ma et al.**	low	low	low	low	high	low	low
**Fevrier et al.**	low	low	low	low	low	low	low
**Benson et al.**	low	low	low	low	low	low	low
**Sada et al.**	low	low	low	unclear	high	low	low
**Zand et al.**	high	low	low	unclear	high	low	low
**Ananthakrishnan et al.**	low	low	low	unclear	high	low	low

Tables S3: ROBINS-I.

Author	D1	D2	D3	D4	D5	D6	D7	Overall
**Kong et al.**	high	high	low	low	low	low	low	moderate
**Lahat et al.**	high	high	low	low	low	low	low	moderate
**Benedicenti et al.**	moderate	high	low	low	low	low	low	moderate
**Zhou et al.**	high	high	moderate	low	unclear	low	low	high
**Choo et al.**	low	low	low	low	low	low	low	low
**Huo et al.**	moderate	high	moderate	low	low	low	low	moderate
**Lim et al.**	moderate	high	moderate	low	unclear	low	low	moderate
**Lahat et al. (2023)**	low	moderate	low	low	low	low	low	low
**Atarere et al.**	moderate	high	moderate	low	unclear	low	low	moderate
**Rammohan et al.**	moderate	high	moderate	low	low	moderate	low	moderate
**Pereyra et al.**	moderate	low	low	low	low	low	low	low
**Peng et al.**	low	low	low	low	low	low	low	low
**Yeo et al.**	low	high	low	low	low	low	low	low
**Gorelik et al.**	moderate	high	moderate	low	low	low	low	moderate

**Samaan et al.**	low	high	low	low	unclear	low	low	moderate
**Cankurtaran et al.**	high	high	low	low	low	low	low	moderate
**Wagholikar et al.**	low	low	low	low	low	low	low	low
**Sciberras et al.**	moderate	high	low	low	low	low	low	moderate
**Gravina et al.**	low	high	moderate	low	low	low	low	moderate
**Pradhan et al.**	moderate	moderate	moderate	low	unclear	low	low	moderate
**Wang et al. (2024)**	moderate	moderate	low	low	low	low	low	moderate

Abbreviations:

- D1: Bias due to confounding.
- D2: Bias in selection of participants into the study.
- D3: Bias in classification of interventions.
- D4: Bias due to deviations from intended interventions.
- D5: Bias due to missing data.
- D6: Bias in measurement of outcomes.
- D7: Bias in selection of the reported result.

Tables S4: Journals, quartiles and SJR's of the included studies.

Author	journal	Q	SJR
**Kong et al.**	Helicobacter	1	1.04
**Schneider et al.**	eClinicalMedicine	1	3.52
**Lahat et al.**	Diagnostics	2	0.67
**Benedicenti et al.**	Gastroenterology Report	2	0.86
**Truhn et al.**	the journal of pathology	1	2.43
**Zhou et al.**	International Journal of Interactive Multimedia and Artificial Intelligence	1	0.9
**Choo et al.**	ANZ Journal of Surgery	2	0.45
**Huo et al.**	Health and Technology	2	0.62
**Imler et al.**	Clinical Gastroenterology and Hepatology	1	3.09
**Lim et al.**	Clinical Gastroenterology and Hepatology	1	3.09
**Imler et al.**	Clinical Gastroenterology and Hepatology	1	3.09
**Bae et al.**	JMIR Medical Informatics	2	0.95
**Denny et al.**	Medical Decision Making	1	1.17
**Li et al.**	Cancer Medicine	1	1.17
**Lahat et al.**	Scientific Reports	1	0.9
**Laique et al.**	Gastrointestinal Endoscopy	1	1.75
**Blumenthal et al.**	Journal of General Internal Medicine	1	1.73
**Harkema et al.**	Journal of the American Medical Informatics Association: JAMIA	1	2.12
**Raju et al.**	Gastrointestinal Endoscopy	1	1.75
**Nayor et al.**	Digestive Diseases and Sciences	1	1.07
**Atarere et al.**	Digestive Diseases and Sciences	1	1.07

**Seong et al.**	BMC Medical Informatics and Decision Making	1	1
**Lee et al.**	Journal of Clinical Gastroenterology	2	0.91
**Denny et al.**	Journal of the American Medical Informatics Association: JAMIA	1	2.12
**Parthasarathy et al.**	Gastrointestinal Endoscopy	1	1.75
**Rammohan et al.**	Cuerues	NA	NA
**Pereyra et al.**	Journal of Clinical Gastroenterology	2	0.91
**Song et al.**	Journal of Clinical Medicine	1	0.88
**Peng et al.**	Scientific Reports	1	0.9
**Tinmouth et al.**	Gastrointestinal Endoscopy	1	1.75
**Mehrotra et al.**	Gastrointestinal Endoscopy	1	1.75
**Becker et al.**	International Journal of Medical Informatics	1	1.11
**Hou et al.**	Digestive Diseases and Sciences	1	1.07
**Wang et al.**	Frontiers in Artificial Intelligence	2	0.76
**Yeo et al.**	Clinical and molecular hepatology	1	3.13
**Gorelik et al.**	Gastrointestinal Endoscopy	1	1.75
**Samaan et al.**	Arab journal of gastroenterology	3	0.34
**Cankurtaran et al.**	Cuerues	NA	NA
**Wenker et al.**	Clinical Gastroenterology and Hepatology	1	3.09
**Imler et al.**	Gastrointestinal Endoscopy	1	1.75
**Li et al.**	Clinical Gastroenterology and Hepatology	1	3.09
**Li et al.**	eClinicalMedicine	1	3.52
**Wagholikar et al.**	Proceedings - 2012 IEEE 2nd Conference on Healthcare Informatics, Imaging and Systems Biology	NA	NA
**Sciberras et al.**	Journal of Crohn's & colitis	1	2.71
**Sherman et al.**	Hepatology communications	1	2.22
**Stidham et al.**	Inflammatory bowel diseases	1	1.55
**Ganguly et al.**	Journal of clinical gastroenterology	2	0.91

**Van Vleck et al.**	International journal of medical informatics	1	1.11
**Ma et al.**	Surgical Endoscopy and Other Interventional Techniques	1	1.12
**Gravina et al.**	Digestive and Liver Disease	2	0.87
**Fevrier et al.**	Journal of Medical Systems	1	0.97
**Benson et al.**	JCO clinical cancer informatics	1	1.4
**Sada et al.**	Medical Care	1	1.38
**Zand et al.**	Journal of Medical Internet Research	1	2.02
**Ananthakrishnan et al.**	Inflammatory bowel diseases	1	1.55
**Pradhan et al.**	Hepatology communications	1	2.22
**Wang et al.**	Journal of gastroenterology and hepatology	1	1.18

Abbreviations: Q: Quartile | SJR: SCImago Journal Rank
